# Food Marketing and Power: Teen-Identified Indicators of Targeted Food Marketing

**DOI:** 10.3390/ijerph19137815

**Published:** 2022-06-25

**Authors:** Charlene Elliott, Emily Truman, Nikki Stephenson

**Affiliations:** 1 Department of Communication, Media and Film, University of Calgary, Calgary, AB T2N 1N4, Canada; emily.truman@ucalgary.ca; 2 Department of Community Health Sciences, Cumming School of Medicine, University of Calgary, Calgary, AB T2N 1N4, Canada; nstephe@ucalgary.ca

**Keywords:** food, marketing, advertising, youth, teenager, adolescent, monitoring, policy, public health, power

## Abstract

Food marketing is powerful and prevalent, influencing young people’s food attitudes, preferences, and dietary habits. Teenagers are aggressively targeted by unhealthy food marketing messages across a range of platforms, prompting recognition of the need to monitor such marketing. To monitor, criteria for what counts as teen-targeted food marketing content (i.e., persuasive techniques) must first be established. This exploratory study engaged teenagers to explore the “power” of food marketing by identifying what they consider to be teen-targeted marketing techniques within various food marketing examples. Fifty-four teenagers (ages 13–17) participated in a tagging exercise of 19 pre-selected food/beverage advertisements. Assessed in light of age and gender, the results showed clear consistency with what indicators the participants identified when it comes to selecting “teen-targeted” ads—with advertisements most frequently chosen as “teen-targeted” containing humor (particularly irony) and celebrities. When it comes to specific indicators used by teenagers, visual style dominated, standing as the marketing technique with the most “power” for teenagers. The findings shed much needed insight into the elements of power—and more precisely, the specific marketing techniques persuasive to teenagers—which are necessary to inform monitoring efforts and to create evidence-based policy.

## 1. Introduction

In February 2022, the World Health Organization (WHO) released its latest report on food marketing to young people—a narrative review on the nature, extent, and impact of food marketing on children and adolescents [1]. Along with providing an up-to-date review of the research literature on this topic, the report aimed to support the development of policy guidelines that will help countries implement “effective actions” to shield young people from the negative effects of food marketing ([1] p.1). The report’s conclusions were succinct: food marketing remains “pervasive and persuasive” ([1] p.16). More specifically, food marketing promotes unhealthy foods, targets young audiences, and uses a range of persuasive techniques designed to resonate with them.

The 2022 WHO report joins the ever-expanding corpus of literature that pinpoints food marketing as a critical element of the food environment, an element that influences young people’s food attitudes, preferences, and dietary habits [2]. “Big Food”, of course, has long been critiqued for promoting food of dubious nutritional value to consumers of all ages [3,4,5], and for creating an “eat more” environment [6] that urges consumers to eat larger quantities and more often. Recent exposés of the food industry have further revealed how manufacturers manipulate foods to elicit addictive behaviors by tapping into, and exploiting, our biological preference for sweetness [7]. However, the problem of food marketing to children has been given especial attention by national and international health bodies/organizations. One common thread within this literature focused on children is a call to action, not only in terms of creating policy to foster an “enabling food environment” for children [8], but also in building a strong monitoring framework to ensure the policy objectives are being met [8].

Such calls for effective policy and monitoring related to unhealthy food marketing to children have, in recent years, acknowledged the need to consider the teen audience as well. This stems from growing evidence that teenagers are aggressively targeted by food marketers across the full spectrum of communicative platforms [9]—from television [10], websites [11], and online video platforms [12] to social media [13,14,15,16] and within the built environment. Cultural spaces for teenagers are riddled with persuasive food marketing messages [9,12], which—given their powerful emotional and social appeals—teenagers are generally unable or unwilling to resist ([17] p.29). This persuasiveness and ubiquity of food marketing matters, not merely in terms of immediate consumption, but also over the longer term, as it generates behavioral and taste transformations in ways not immediately apparent. As Kelly et al.’s [18] conceptual model outlines, a hierarchy of unhealthy food promotion effects exists, whereby individual exposures to food marketing (and its persuasive power) prompts a “sequenced set of effects” linked to “weight related outcomes” [18]. In particular, food marketing creates awareness of products and brands, positively influences attitudes and preferences, and drives purchase intent and consumption. Notably, this model does not posit a simple “cause-effect” between food marketing exposure and obesity; rather, it draws attention to the need to recognize “the cumulative effects of exposure, the permanence of effects over the longer term, and the impacts of integrated campaigns that span multiple media” ([18] p.e94). Viewed in this light, food marketing messages become more than promotional vehicles; they are part and parcel of the social, environmental, and behavioral factors that impact teenagers’ health potential—moving from adolescence into adulthood.

In short, the power and ubiquity of teen-targeted food marketing, coupled with teenagers’ vulnerability to marketing appeals, has heightened awareness of the need for monitoring. In Canada, the notion of monitoring food marketing to teenagers is part of the federal government’s Healthy Eating Strategy [19], whereby Health Canada is working to foster supportive environments for healthy eating and has (for the first time) also advised Canadians to “be aware of food marketing” in Canada’s Food Guide. However, to monitor food marketing to teenagers, clear criteria for what counts as teen-targeted food marketing must first be worked out. 

This, however, is no simple task. One challenge with the existing literature on teenagers and food marketing is that very little of it engages with the actual content of the marketing message. With the exception of a few studies [9,20], the specific persuasive techniques used within the advertisements are glossed over. To date, only one study [9] engages with the persuasive techniques found within marketing found across a spectrum of platforms. Moreover, a scoping review of the literature on teenagers and food marketing revealed a lack of consistency in the indicators used to identify it [21] and high degrees of subjectivity (i.e., based on the interpretation of the researchers). What is needed is more insight into teen perspectives on what makes food marketing teen-targeted and why. 

In light of this research gap, our study engaged teenagers to explore the “power” (or persuasive techniques) of food marketing [8]. In particular, we invited teenagers to review examples of food marketing from a variety of platforms and to consider whether they were teen-targeted. If so, the teenagers identified the techniques that (from their perspective) made the specific advertisement teen-targeted. These answers were assessed in light of age and gender, with an eye to revealing the consistency and nuances in both identifying teen-targeted food marketing and techniques (or indicators) within such marketing. Such indicators matter because they are the specific elements that made an advertisement “teen-targeted” to the teenagers consulted. The findings shed much needed insight into the elements of power—and more precisely, the specific marketing techniques persuasive to teenagers—which are necessary to inform monitoring efforts and to create evidence-based policy.

## 2. Methods

### 2.1. Recruitment and Procedure

Teenagers (ages 13–17) were recruited through the website of the primary investigator, personal networks of research team members, and by word-of-mouth between January and May 2021. The study was approved by the University of Calgary Conjoint Faculties Research Ethics Board (REB19-0020) and conducted online, via Zoom, due to COVID-19 restrictions regarding in-person meetings. The participants were given verbal and written details about the study aims and procedures and provided written consent to participate. On this consent form, they were able to self-identify gender (girl/boy/gender non-conforming).

In the study, the teenagers were asked to participate in a tagging exercise of 19 pre-selected food/beverage advertisements. The selected advertisements depicted a variety of products and represented a variety of formats (e.g., digital images, video, animated GIFs), from a variety of platforms (e.g., YouTube, Instagram, magazines). Table 1 details the product, platform, and content of the food/beverage marketing examples reviewed by the teen participants.

The 19 food marketing examples were shown to teenagers one at a time (in a PowerPoint slide show). Following the display of each image, a polling question appeared on each participant’s screen, stating: “If you think this is an example of teen-targeted food marketing, please select all of the tags that apply to describe why it is teen-targeted from the list below.” Listed tags included: *animated character, teenaged actor, celebrity, language, music, humor, special offer, visual style, theme,* or *other (you tell us!)*. When “other” was selected, the participants were asked to explain in more detail by typing in the chat window. Importantly, the participants were instructed to leave the poll question blank if they did not think the advertisement was specifically teen-targeted. The list of evidence-based tags (or indicators) was drawn from a scoping review on teen food marketing literature [16] and subsequently validated in follow-up focus groups with 18 teenagers conducted by the same authors.

Since one aim of the study was to investigate the consistency and nuances in both identifying teen-targeted food marketing and indicators within such marketing, not all of the examples reviewed by the teenagers were clearly teen-targeted. Some were explicitly teen-targeted, others were not, and some were in the grey zone (see Table 2). In addition to the teenage subjectivity in determining whether an advertisement was “teen-targeted” (which this study sought to probe), each food marketing example also had certain boundaries or nonsubjective elements when it came to the applicability of indicators. For instance, one of the food marketing examples reviewed by the teenagers was a package of Kraft Dinner (boxed macaroni and cheese). The package was blue with “KD” written in orange styled font. Since the KD package did not contain indicators such as an *animated character, teenaged actor, celebrity, music,* or *special offer*, these “tags” should not be selected. As such, we identified the *range* of indicators that might reasonably be selected according to the content of each advertisement.

### 2.2. Statistical Analysis

All statistics were completed using Stata 16 IC. Food marketing content (i.e., tags) and self-identified gender were assessed as categorical variables. Age was assessed as both a continuous variable as well as a categorical variable (≤14 and >14). Indicator variables were created to determine frequencies for each tag option. Univariate analyses of study design characteristics were calculated with Clopper–Pearson 95% confidence intervals. Correlation between each of the potential tag categories was assessed using Pearson’s correlation coefficients. Further bivariate analyses of indicators for teen targeting stratified by food marketing content (i.e., tags) and study characteristics (i.e., gender, age), as well as comparisons between tag selection of specific images, were assessed via cross-tabulation and post hoc calculation of Clopper–Pearson 95% confidence intervals or two-sample tests of proportions (significance determined using *p* < 0.05). Association via logistic regression between teen-indicated teen-targeted advertisements and researcher-indicated teen-targeted advertisements by tag category via logit included both binary gender and continuous age variables as confounding factors. The probability of tag selection within this regression model and the 95% confidence interval calculated using the standard error of the linear prediction were calculated using the predict post-estimation tools for logit. Where applicable, estimates were interpreted as stratum specific linear combinations of the regression coefficients.

## 3. Results

Fifty-four teenagers took part in the study, with a mean age of 14.4. Slightly more girls than boys participated (54% vs 46%) (see Table 3); no participants self-identified as gender non-conforming.

### 3.1. Teen-Targeted Food Marketing Examples: Frequency of Identification 

Table 4 shows the breakdown of advertisements identified as “teen-targeted” by the teenagers. Their categorization (as teen-targeted or not) generally aligned with the researchers’ categorization detailed in Table 2. However, some participants identified food marketing examples (such as Goldfish Crackers and Kraft Dinner packaging) which were not considered teen-targeted by the researchers as “teen-targeted”—the reasons the teens provided will be addressed shortly. Food marketing featuring cheeky humor and celebrities (such as those found in Pop Tarts, Sprite, and Pop Chips) were most frequently chosen as teen-targeted, whereas packaging examples (Pringles potato chips and Kraft Dinner) were selected the least. Recall that the teens were instructed to identify whether the food marketing/advertisement was teen-targeted; as such, this task was about the power of the marketing, not about whether teens thought the food itself was for teenagers. 

### 3.2. Teen-Targeted Indicators: Frequency of Identification

**Figure 1:**
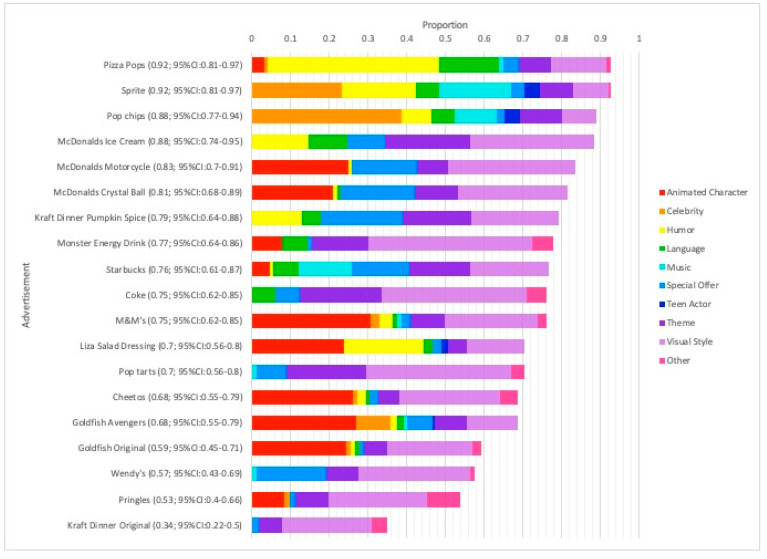
Stacked bar graph showing the relative contribution of each tag to the proportion of indicators for identifying an instance of teen-targeted food marketing.

When it comes to specific indicators used by the teenagers, *visual style* (0.44; 95% CI: 0.41–0.47) was the most frequently selected, followed by *animated character* (0.21; 95% CI: 0.19–0.24) and *theme* (0.21; 95% CI: 0.19–0.24). Figure 1 depicts the relative contribution of each tag to the proportion of total indicators selected by the teens for each instance of food marketing. In terms of the advertising examples most frequently classified as “teen-targeted” by the participants (i.e., promotions for Pizza Pops and Sprite), Figure 1 shows the variety of tags selected (9 of 10 options for both) but also the *consistency of identification* among teenagers when it comes to the tags commonly selected (i.e., *humor*, *language*, and *visual style* for Pizza Pops (0.80; 95% CI: 0.67–0.88, 0.28; 95% CI:0.17–0.41, and 0.26; 95% CI:0.16–0.39 respectively); and *celebrity*, *humor*, and *music* for Sprite (0.85; 95% CI: 0.73–0.92, 0.70; 95% CI: 0.57–0.81, and 0.69; 95% CI: 0.55–0.79)).

### 3.3. Teen-Targeted Indicators: Frequency of Identification by Gender and Age

Statistical differences existed when it came age and the selection of certain indicators (see Table 5). Younger teenagers (ages 13–14) were more likely than older teenagers (age 15) to select *animated character* as an indicator; they also selected the *language* “tag” less frequently than older teens did. Significant differences were also found between the 13- and 14-year-olds when it came to *humor* as an indicator, since the youngest teens selected *humor* as an indicator less frequently withing the “teen-targeted” advertising. *Special offer* was identified more frequently by 16-year-olds than by 13-year-olds. Boys also selected the *special offer* indicator more frequently than girls did.

Taken together, Table 4 and Table 5 and Figure 1 above show distributional differences in the probability of indicator selection by food marketing content, gender, and age; these factors were included as covariates for the logistic regression modelling in the following section.

### 3.4. Teen-Targeted Indicators: Probability of Identification by Age and Gender

As detailed in the methods, the researchers used pre-established indicators of teen-targeted advertising [21] to categorize the 19 food advertisements as “teen-targeted”, “not teen-targeted”, or falling into a “grey-zone” (see Table 2).

**Figure 2:**
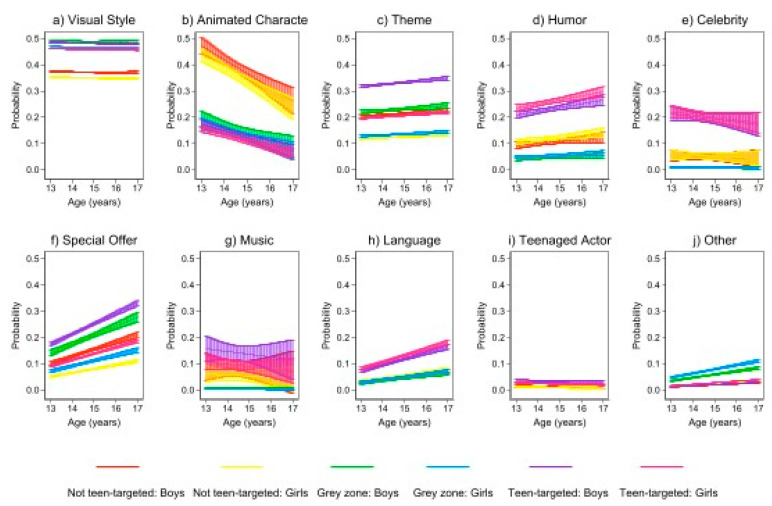
The probability of selecting teen-targeted indicators by gender and age.

Overall, teens were likely to tag *visual style* across all advertisements, irrespective of how researchers categorized it (i.e., the advertisements the researchers categorized as teen-targeted, not teen-targeted, or grey-zone were tagged by the participants as teen-targeted due to visual style by 47%, 48%, and 35% of the teens, respectively). In contrast, *theme* (26%), *humor* (23%), *celebrity* (18%), *special offer* (17%), *music* (15%), *language* (11%), and *teen actor* (2%) were all more likely to be identified in the advertisements the researchers (and teens) categorized as “teen-targeted”. The only outlier was *animated* character, which was identified most consistently (15%) in the ads that the researchers categorized as “not teen-targeted”. This proves interesting insomuch as animated character was the second most selected indicator overall in this study but was most frequently chosen in relation to advertisements that the researchers did not consider to be teen-targeted. (N.B. The researchers considered animated characters in certain advertisements to be kid-appealing but not teen-targeted; the participants’ responses indicated that some of them viewed this differently.) Figure 2 shows the probability of the teens’ tag selection within each of the three researcher-defined categories, examined by indicator.

Supplementary File S1 provides a detailed analysis of the probability of participants selecting each indicator, adjusted for product, gender, and age. In terms of topline observations, however (and when adjusted for marketing content and age), the probability of selecting the *visual style* tag was the highest among examples that the researchers categorized as both teen-targeted and in the grey zone (0.47; 95% CI: 0.43–0.52 and 0.48; 95% CI: 0.42–0.54, respectively; see Figure 2, Panel a). Age did not influence the probability of tag selection, but boys were more likely to identify *visual style* in ads categorized as teen-targeted compared to the grey-zone/not teen-targeted ads. In contrast, the probability of selecting *animated character* as a tag was the highest among advertising examples considered not teen-targeted by the researchers (0.32; 95% CI: 0.27–0.37) and lower among teen-targeted and grey-zone examples (0.15; 95% CI: 0.11–0.19) (see Panel b). The probability of selecting the animated character tag did not differ by gender but did decrease with age.

*Theme*, like *visual style,* also had a gendered dimension. Boys had a consistently higher probability of selecting *theme.* For all participants, the probability of selecting *theme* as an indicator was the highest among advertising examples categorized as teen-targeted by researchers (0.26; 95% CI: 0.21–0.30) and lower for not teen-targeted and grey-zone examples (0.17; 95% CI: 0.13–0.21 and 0.17; 95% CI: 0.13–0.22, respectively). 

The probability of selecting the *humor tag* did not vary by gender or age. However, the marketing examples categorized as teen-targeted by the researchers were most likely to be tagged with humor (0.23; 95% CI: 0.19–0.27) (not teen-targeted or grey-zone examples: 0.13; 95% CI: 0.09–0.18; and 0.05; 95% CI: 0.02–0.07, respectively).

When adjusted for marketing content, gender, and age, the probability of selecting the *special offer* tag was the highest among marketing examples the researchers categorized as teen-targeted (0.17; 95% CI: 0.13–0.20) compared to those in the grey zone (0.14; 95% CI: 0.10–0.18), and not teen-targeted categorized content (0.11; 95% CI: 0.07–0.15). The probability of selecting *special offer* increased with age and was consistently higher for boys than for girls. Finally, the probability of selecting the *language* tag was the highest among marketing examples considered to be teen-targeted (0.11; 95% CI: 0.08–0.13) and the lowest for not teen-targeted and grey-zone categories (0.05; 95% CI: 0.03–0.08 and 0.04; 95% CI: 0.02–0.06, respectively). The probability of selecting *language* as a tag increased with age across all researcher categories. 

## 4. Discussion

This exploratory study breaks new ground in engaging teenagers to identify the “power” of selected food marketing examples across a variety of platforms in order to assess consistency of that identification (and in light of age and gender). Such a project is helpful to monitoring efforts, given that the literature on teen engagement with food marketing content is still very much in its infancy. While some recent studies productively explore teenager engagement with food brands and marketing [11,13,14], they sidestep the important issue of advertising content as a key factor driving engagement. To date, only two published studies [9,20] focus in depth on the various persuasive techniques within food advertisements that attract teenagers’ attention and make the message meaningful. Only one of those studies [9] considers food marketing across a variety of platforms.

Although monitoring food marketing to teenagers is positioned as simply a technical issue, identifying the persuasive power (specific techniques or indicators) within this marketing is far more complex. The 54 teenagers in this study provided fascinating insight into what engaged their attention when it comes to food marketing messages, with general alignment in terms of what teenagers identify as teen-targeted food marketing (compared to not teen-targeted) based on particular indicators. When certain exceptions arose with respect to what participating teenagers selected as “teen-targeted”—as per the case in which some teenagers identified food marketing examples like Goldfish Crackers and Kraft Dinner—it appears that these teens were conflating the marketing with the food itself. That is, these teenagers (presumably) were classifying the *food* as teen food rather than focusing on the packaging: this move was borne out by the respondent who selected the image of a package of Kraft Dinner macaroni and cheese as teen-targeted, chose “other” as an indicator with the explanation that “it is easy to make.” Ease of preparation is not about the appeals on the actual package and does not pertain to the marketing. Rather, it is a functional attribute of Kraft Dinner. Here, it is worth noting that qualitative research with Canadian teenagers reported that these two products—Kraft Dinner and Goldfish Crackers—were given as specific examples of “teen-food” by the participants [22]. In particular, participants explained their classification by connecting the functional attributes of these two food products to teenagers’ unique characteristics and concerns as teenagers. Kraft Dinner was deemed teen food because it was easy to make (and “most teens are lazy when it comes to making food”) [22]. Goldfish Crackers were considered teen food because they are both inexpensive (and “teenagers are usually broke”) and plentiful to share with friends [22].

When it comes to marketing power, the specific appeals that capture teenagers’ attention, this study reveals that visual style dominated. Visual style is a term used to capture aspects such as color, font, or animated effects, which contribute to the overall “feel” of an advertisement. Visual style was the most frequently selected indicator and the tag most used across *all* advertisements (i.e., including those the researchers categorized as not teen-targeted or grey-zone). Notably, visual style was also the top indicator reported in a participatory study where teens captured their own examples of “teen-targeted” food marketing in real-world settings and identified what made them “teen-targeted” [9]. Here, teens used visual style to describe one of every two advertisements they captured [9]. Other research on food marketing power similarly observes the importance of “appearance-related features” to teenagers ([20] p.9 )and the “highly appealing” nature of visual style for teens [23]. Indeed, the persuasive appeal of visual style extends to a range of consumer products beyond the realm of food [24,25].

*Visual style* is a highly salient element of marketing power for teenagers. However, alongside this frequently used indicator of “teen-targeted” food marketing found in our study sit several others, such as *animated character* and *theme*. These findings reveal important nuances with respect to *age* and relative power, since it were the younger teenagers who were more likely to select *animated character* as an indicator of teen targeted food marketing. The waning appeal of cartoon characters as children age has been documented in experimental [26,27] and qualitative research studies [28], and is an important consideration in terms of how marketing power fluctuates depending on the audience. In the same vein, our study showed that the probability of selecting *language* and *special offer* increased with age, revealing the shifting priorities of teenagers in terms of what proved persuasive with them. 

Finally, our study revealed the importance of engaging teenagers in studies that aim to inform policies directly relevant to them (such as monitoring food marketing to teenagers) while highlighting indicators most useful to monitoring efforts. Recall that the participants were instructed to consider whether the *food marketing/advertisement* was teen-targeted and if so, to indicate what made it teen-targeted. Teenagers only selected indicators for the marketing they thought was teen-targeted. While the results showed considerable alignment in terms of what teens identified as teen-targeted food marketing, some advertisements *not* considered teen-targeted by the researchers were flagged as such by the participants. This was particularly the case for examples containing cartoon characters that the researchers considered child-appealing but not teen-targeted—and so they were not categorized as teen-targeted by the researchers. Fifteen percent of teens flagged these as teen-targeted. While this percentage is not high, it underscores the differing subjectivities at play between adults and teens when it comes to interpretating “teen-targeted.” 

Most significantly, the study revealed strong consistency among participants when it came identifying *why* various advertisements were teen-targeted: indicators such as *theme, humor, celebrity, special offer, music, language*, and *teen actor* were all more likely to be identified in the marketing examples categorized as teen-targeted. The importance of these indicators—especially alongside visual style, discussed above—is mirrored in recent research where teenagers engage with capturing and tagging teen-targeted food marketing in real-world settings [9]. 

### Strengths and Limitations 

This was an exploratory study, designed to probe the perceptions of teen-specific or teen-targeted persuasive power within food marketing. Study strengths include the focus on an understudied topic, providing marketing examples appealing to various audiences and across a full range of platforms to assess, a comprehensive set of indicators for teens to select from; the option for teens to include indicators, justifications that were salient to them (using an “other” category and a free text form field), consideration of age and gender, and policy relevance. Limitations stem from the exploratory nature. Future studies with a larger number of teenagers across the age range would be useful. 

## 5. Conclusions

Food marketing is powerful and prevalent, influencing young people’s food attitudes, preferences, and dietary habits. Given calls to monitor such marketing to teenagers, a closer look at the content of the advertising is required. Findings from this study provide much needed insight into the elements of power—and more precisely, the specific marketing techniques persuasive to teenagers. Such insight is necessary to inform monitoring efforts and to create evidence-based policy, particularly since a “one-size fits all” approach—one that treats child- and teen-targeted indicators as synonymous—is not appropriate. Teenagers are not the same as children, nor are they targeted with the same appeals: indeed, there is little purchase in seeking to explore the persuasive appeals of food marketing from a teen perspective if these teen-identified appeals are glossed over by those creating policy and leading monitoring efforts. Moreover, when viewed in light of the *hierarchy of unhealthy food promotion effects* model [18], it becomes clear that knowing these indicators is no trivial matter. These persuasive elements comprise the power of an individual food advertisement, combining to impact food attitudes, preferences, and dietary practices [1,2].

## Figures and Tables

**Table 1:** Food marketing examples selected for teen-targeted indicators testing.

Food Brand/Product	Format and Original Platform	Description of Content
Popchips	2D print ad, magazine	Image of pop singer Katy Perry in a blue dress (against a blue background), holding up a bag of original Popchips in each hand at chest level. Main caption reads: “Nothing fake about ‘em”.
Pizza Pops	Video (6 s), Instagram	Adult male in pizza pop costume hands out samples on the street, then spins a sign that reads “Bake at 420”
Wendy’s	GIF (3 s), Instagram	Stop-motion image of loose French fries moving into a shape that represents “$1”.
McDonalds McRib I (Motorcycle)	GIF (10 s), Instagram	Silhouette of motorcycle rider against brightly coloured striped background, ends with message “McRib is back. 29 October–25 November 2018”.
McDonalds McRib II (Crystal Ball)	GIF (10 s), Instagram	Silhouette of fortune teller, crystal ball with multiple messages and images: “it’s coming”; cartoon image of McRib sandwich; cartoon of cellphone with McRib finder map.
Liza Salad Dressing	2D print ad, magazine	Image of large cartoon chicken attempting to catch a frisbee in a park, humans in background. In bottom right-hand corner a very small caption reads “Make new friends, eat more salad” next to an image of a salad dressing bottle.
Sprite	Video (60 s), YouTube	NBA player LeBron James walks through a large house and yard (with teen actors in the background, lounging and dancing, and rapper Lil Yachty); repeatedly claims he is not telling the audience to drink Sprite.
Coca-Cola	2D image (6 images in 2 × 3 grid), Instagram	Image grid features similarly designed red and black stylized artwork featuring Coke bottle/can, hearts, and motivational sayings (i.e., “Love is the standard”, “Happiness looks good on you”).
Pop Tarts	2D image (6 images in 2 × 3 grid), Instagram	Image grid features photo realist pictures of summer themes (i.e., fireflies, s’mores) in both dark/moody and bright colourways, some including product packaging.
Pringles	2D image, food package	Photo of Pringles Canister, Sour Cream and Onion flavor.
Cheetos	2D image, food package	Photo of Cheetos Puffs bag.
M&M’s	2D image, food packaging	Photo of M&M’s Chocolate Bar.
Goldfish Crackers Original	2D image, food package	Photo of Goldfish Crackers bag, Original flavor.
Goldfish Crackers Avengers	2D image, food package	Photo of Goldfish Cracker bag, featuring cartoon images of Avengers characters.
Monster Energy Drink	2D image, food package	Photo of Monster Punch can, featuring stylized skull imagery.
McDonalds Ice Cream Cone	2D image, Sign/poster	Image of a McDonald’s ice cream cone with a charge cord plugged into the bottom of it, held up by a disembodied hand. Text reads: “Get fully charged. Download the new app”.
Starbucks	Video (15 s), YouTube	Highlights three specialty drink flavors in holiday cups, in a winter setting (snowman, blowing snow, snow drifts with drinks set in them).
Kraft Dinner Original	2D image, food packaging	Photo of Kraft Dinner box, Original flavor.
Kraft Dinner Pumpkin Spice	2D image, Twitter	Image of fall setting (fallen leaves, pumpkins) with disembodied hand holding a takeout drink cup (branded with KD logo) filled with Kraft Dinner macaroni and cheese. Text reads: “Pumpkin Spice KD. Coming this October”.

**Table 2:** Examples of food marketing used in the study (based on presence of teen-targeted indicators in the content, as defined by the researchers).

Teen-Targeted	Not Teen-Targeted	Grey Zone
Pizza Pops Sprite Pop Chips McDonald’s Ice Cream Cone McDonald’s McRib I McDonald’s McRib II Coca-Cola Pop Tarts	Starbucks M&M’s Chocolate Bar Liza Salad Dressing Goldfish Crackers Avengers Goldfish Crackers Original Kraft Dinner Original	Kraft Dinner Pumpkin Spice Monster Energy Drink Cheetos Wendy’s Pringles

**Table 3:** Participant characteristics.

Number of Participants	
Gender	
Girl	29
Boy	25
Age	
13 years	11
14 years	19
15 years	16
16 years	6
17 years	2

**Table 4:** The proportions of teens identifying the examples of food marketing as teen-targeted.

Marketing Example	Indicated as Teen-Targeted	Proportion (95% CI)
Pizza Pops	n = 50/54	0.93 (0.82–0.97)
Sprite	n = 50/54	0.93 (0.82–0.97)
Popchips	n = 48/54	0.89 (0.77–0.95)
McDonald’s Ice Cream Cone	n = 38/43	0.88 (0.75–0.95)
McDonald’s McRib I	n = 45/54	0.83 (0.71–0.91)
McDonalds McRib II	n = 44/54	0.81 (0.69–0.90)
Kraft Dinner Pumpkin Spice	n = 34/43	0.79 (0.64–0.89)
Monster Energy Drink	n = 42/54	0.78 (0.65–0.87)
Starbucks	n = 33/43	0.77 (0.62–0.87)
Coke	n = 41/54	0.76 (0.63–0.85)
M&M’s	n = 41/54	0.76 (0.63–0.85)
Liza Salad Dressing	n = 38/54	0.70 (0.57–0.81)
Pop-Tarts	n = 38/54	0.70 (0.57–0.81)
Cheetos	n = 37/54	0.69 (0.55–0.79)
Goldfish Crackers Avengers	n = 37/54	0.69 (0.55–0.79)
Goldfish Crackers Original	n = 32/54	0.59 (0.46–0.71)
Wendy’s	n = 31/54	0.57 (0.44–0.70)
Pringles	n = 29/54	0.54 (0.40–0.66)
Kraft Dinner Original	n = 15/43	0.35 (0.22–0.50)

**Table 5:** Frequency of teen-targeted indicators identified by gender and age (statistical differences determined by exclusivity of the 95% confidence intervals are bolded and denoted by *).

**Characteristic**	**Observations**	Animated Character % (95% CI)	Celebrity % (95% CI)	Humor % (95% CI)	Language % (95% CI)	Music % (95% CI)	Special Offer % (95% CI)	Teenaged Actor % (95% CI)	Theme % (95% CI)	Visual Style % (95% CI)	Other % (95% CI)
**GENDER**											
**Boys (n = 25)**	n = 451	22.17 (18.57–26.25)	10.18 (7.71–13.33)	14.19 (11.26–17.73)	6.87 (4.87–9.61)	8.43 (6.19–11.37)	19.29 (15.90–23.20) *	1.77 (0.89–3.51)	26.61 (22.73–30.88) *	45.01 (40.47–49.64)	2.66 (1.52–4.63)
**Girls (n = 29)**	n = 531	20.15 (16.95–23.78)	10.57 (8.22–13.49)	16.01 (13.13–19.38)	7.72 (5.73–10.32)	5.65 (3.98–7.97)	10.17 (7.87–13.05) *	1.32 (0.63–2.74)	16.01 (13.13–19.38) *	42.75 (38.60–47.01)	3.58 (2.29–5.54)
**AGE**											
**13 years (n = 11)**	n = 209	**25.36** (19.92–31.70) *	8.13 (5.11–12.70)	9.57 (6.25–14.37) *	**4.31** (2.25–8.07) *	7.18 (4.37–11.57)	9.09 (5.87–13.82) *	1.44 (0.46–4.36)	20.57 (15.62–26.60)	44.98 (38.36–51.78)	1.91 (0.72–4.99)
**14 years (n = 19)**	n = 361	**25.24** (20.75–30.32) *	14.51 (11.04–18.84)	18.61 (14.70–23.29) *	**4.73** (2.87–7.70) *	7.57 (5.12–11.05)	13.25 (9.94–17.45)	1.89 (0.85–4.15)	17.98 (14.13–22.61)	41.64 (36.33–47.15)	2.52 (1.27–4.97)
**15 years (n = 16)**	n = 304	**14.80** (11.23–19.26) *	8.55 (5.89–12.27)	16.45 (12.69–21.05)	**12.50** (9.23–16.72) *	6.91 (4.54–10.37)	17.11 (13.27–21.77)	0.99 (0.32–3.02)	25.33 (20.75–30.53)	45.72 (40.19–51.36)	4.28 (2.50–7.23)
**16 years (n = 6)**	n = 114	21.05 (14.52–29.51)	7.89 (4.16–14.49)	11.40 (6.73–18.66)	7.89 (4.16–14.49)	4.39 (1.84–10.12)	21.93 (15.27–30.46) *	2.63 (0.85–7.85)	17.54 (11.60–25.65)	43.86 (35.04–53.08)	4.39 (1.84–10.12)
**17 years (n = 2)**	n = 38	13.16 (5.58–27.98)	10.53 (4.00–24.92)	18.42 (9.04–33.92)	2.63 (0.37–16.49)	7.89 (2.56–21.82)	7.89 (2.56–21.82)	0	21.05 (10.88–36.80)	39.47 (25.37–55.57)	2.63 (0.37–16.49)

## Data Availability

The anonymized dataset is available from the authors upon request and will be accompanied by a formal data sharing agreement outlining that the data be used for academic purposes only.

## Supplementary Materials

The following supporting information can be downloaded at: https://www.mdpi.com/article/10.3390/ijerph19137815/s1, File S1: Probability Analysis for Each Indicator.
