# Supplementary material for: Food Marketing and Power: Teen-Identified Indicators of Targeted Food Marketing
Source: Int J Environ Res Public Health. 2022 Jun 25;19(13):7815. doi: 10.3390/ijerph19137815 (PMC9265287; doi:10.3390/ijerph19137815)
Supplement: Supplementary file 1 [file ijerph-19-07815-s001.zip › ijerph-1751617-supplementary.pdf]

Probability Analysis for Each Indicator

3.4.1. Visual style

The probability of selecting the *visual style* tag (adjusted for marketing content, gender and age) is highest among examples that the researchers classified as both teen-targeted and "grey-zone" food advertising (0.47; 95% CI: 0.43-0.52 and 0.48; 95% CI: 0.42-0.54, respectively). The probability of selecting the *visual style* tag is slightly lower among examples that are not teen-targeted (0.35; 95% CI: 0.30-0.41). However, if each of these three categories is stratified by age and gender, the advertisements considered *not* teen-targeted differ statistically from the teen-targeted/grey-zone ads. The highest probability of selecting *visual style* tag is with teen-targeted or grey-zone ads. Selection differs significantly by gender: boys are more likely to identify *visual style* as an indicator of teen-targeted compared to the not teen-targeted/grey-zone ads. Overall, the probability of selecting the *visual style* tag is lowest among advertisements considered not teen targeted, with girls having a significantly lower probability than boys (here, age does not influence the probability of tag selection).

3.4.2. Animated Character

The probability of selecting *animated character* as tag, adjusted for marketing content, gender and age, is highest among advertising examples considered not teen-targeted by researchers (0.32; 95% CI: 0.27-0.37), and lower among teen-targeted (0.15; 95% CI: 0.11-0.19) and grey zone examples (0.15; 95% CI: 0.11-0.19). Statistical differences in the probability of selecting animated character as a tag can be seen between advertisements categorized as teen-targeted/grey zone and not teen-targeted. The probability of selecting the animated character tag does not differ by gender, but does decrease with age.

3.4.3. Theme

The probability of selecting *theme* as tag adjusted for marketing content, gender and age, is highest among advertising examples categorized as teen-targeted by the researchers (0.26; 95% CI: 0.21-0.30), and lower in relation to not teen-targeted and grey zone examples (0.17; 95% CI: 0.13-0.21 and 0.17; 95% CI: 0.13-0.22, respectively). Age does not significantly influence the probability of tag selection, but gender does. Boys have a consistently higher probability of selecting *theme*.

3.4.4. Humor

The probability of selecting the *humour* tag, adjusted for marketing content, gender and age, is highest among marketing examples categorized as teen-targeted by researchers (0.23; 95% CI: 0.19-0.27), and lower in relation to not-teen-targeted and grey zone examples (0.13; 95% CI: 0.09-0.18; and 0.05; 95% CI: 0.02-0.07, respectively). These probabilities did not vary by gender or age.

3.4.5. Celebrity

The probability of selecting *celebrity* as an indicator, adjusted for marketing content, gender and age, is highest among marketing examples categorized as teen-targeted (0.18; 95% CI: 0.15-0.22), and lowest in relation to not teen-targeted/grey zone ads (0.08; 95% CI: 0.04-0.11 and 0.01; 95% CI: 0.00-0.11, respectively). However, statistical differences in the probability of selecting *celebrity* as a tag can be seen between those advertisements categorized as not-teen targeted and grey zone from ages 13 to 15. Such differences likely stem from the high representation of those ages within the sample; reasonably, one might expect that if more 17 year-olds participated, significant differences would be seen between all three categories (i.e., teen-targeted, not teen-targeted, grey zone).

3.4.6. Special Offer

When adjusted for marketing content, gender and age, the probability of selecting the *special offer* tag is highest among marketing examples categorized as teen-targeted (0.17; 95% CI: 0.13-0.20), lower the grey zone ads (0.14; 95% CI: 0.10-0.18), and lowest in not teen-targeted ads (0.11; 95% CI: 0.07-0.15). The probability of selecting *special offer* as a tag increased with age, and was consistently higher for boys than girls.

3.4.7. Music

When adjusted for marketing content, gender and age, the probability selected *music* is highest among marketing examples categorized as teen-targeted by researchers (0.15; 95% CI: 0.12-0.17), and lower for the not teen-targeted (0.08; 95% CI: 0.05-0.11), and grey zone examples (0.002; 95% CI: 0.000-0.005). Probabilities did not differ between the three categories or by gender or age. A wide confidence interval exists among those categories researchers deemed to not-be teen-targeted or as teen-targeted indicating increased variance among these groups.

3.4.8. Language

The probability of selecting the *language* tag, adjusted for marketing content, gender and age, is highest among marketing examples considered to be teen-targeted (0.11; 95% CI: 0.08-0.13), and lowest in relation to not teen-targeted (0.05; 95% CI: 0.03-0.08), and grey zone (0.04; 95% CI: 0.02-0.06). Probabilities did not vary between genders; however, the probability of selecting *language* as a tag increased with age for each researcher category.

3.4.9. Teenaged actor

Given that no teenaged actors were present in the advertising examples, the probability of selecting the *teenaged actor* tag, adjusted for marketing content, gender and age, was low for teen-targeted (0.02; 95% CI: 0.01-0.04), and not teen-targeted (0.01; 95% CI: 0.00-0.03). No tags were selected for any of the advertisements within the grey zone. There was no statistical difference seen between any of the researcher categories, and neither age nor gender influenced the predicted probabilities.
